# What Is the Role of Hysteroscopic Surgery in the Management of Female Infertility? A Review of the Literature

**DOI:** 10.1155/2014/105412

**Published:** 2014-03-03

**Authors:** Márcia Mendonça Carneiro

**Affiliations:** Department of Obstetrics and Gynecology, Universidade Federal de Minas Gerais (UFMG), Rua Antonio Torres 186, Sagrada Família, 31030130 Belo Horizonte, MG, Brazil

## Abstract

The position of hysteroscopy in current fertility practice is under debate. There are many randomized controlled trials on technical feasibility and patient compliance demonstrating that the procedure is well tolerated and effective in the treatment of intrauterine pathologies. However, no consensus on the effectiveness of hysteroscopic surgery in improving the prognosis of subfertile women is available. A literature review was performed to explore the available information regarding the role of hysteroscopy in the evaluation and management of female infertility as well as to ascertain evidence that treatment of these uterine abnormalities improves fertility. The debate regarding the role of hysteroscopic surgery in the management of female infertility remains as the published studies did not reach a consensus on the benefit of such an intervention in this setting. The randomized trials do not clearly demonstrate that surgical correction of all intrauterine abnormalities improves IVF outcome. However, published observational studies suggest a benefit for resection of submucosal leiomyomas, adhesions, and endometrial polyps in increasing pregnancy rates. More randomised controlled studies are needed to substantiate the effectiveness of the hysteroscopic removal of suspected intrauterine pathology in women with unexplained subfertility or prior to assisted reproductive technology.

## 1. Introduction

The introduction of hysteroscopy in gynecologic practice revolutionized the diagnosis and treatment of intrauterine disease. New methodological and technological developments have made diagnostic and operative hysteroscopy much more efficient, cost effective, safe, and useful. The most common indication for hysteroscopy is abnormal uterine bleeding (AUB), but it is also used in cases of infertility and Mullerian anomalies [1, 2].

Uterine factors can be found in only 2 to 3% of infertile women, but intrauterine lesions are much more common in this setting (40–50%) [3, 4]. These lesions can compromise spontaneous fertility as well as reduce pregnancy rates in assisted reproduction [3, 4]. Published observational studies suggest increased pregnancy rates after the hysteroscopic removal of endometrial polyps, submucous fibroids, uterine septum, or intrauterine adhesions, which can be found in 10% to 15% of women seeking treatment for subfertility [3].

Evaluation of the uterine cavity is a basic step in female infertility workup. Classically, hysterosalpingography and transvaginal sonography are most commonly used for this purpose. Hysteroscopy, however, is considered the gold standard for diagnosis of intrauterine lesions [2–4]. The benefit of the systematic use of hysteroscopy in the initial assessment of infertility remains unclear and the exploration of the uterine cavity in the initial assessment of infertility should be based on hysterosalpingography (HSG) or hysterosonography. Systematic hysteroscopy before IVF is a widely accepted practice which is supposed to improve pregnancy rates but still lacks scientific evidence. After repeated implantation failure in IVF cycles, uterine cavity should be reevaluated by hysteroscopy and this practice has been demonstrated to improve pregnancy rates [3, 5].

The position of hysteroscopy in current fertility practice is under debate. Although there are many randomized controlled trials on technical feasibility and patient compliance demonstrating that the procedure is well tolerated and effective in the treatment of intrauterine pathologies, there is no consensus on the effectiveness of hysteroscopic surgery in improving the prognosis of subfertile women [6, 7].

A recent review on the effectiveness of hysteroscopy in improving pregnancy rates in subfertile women without other gynecological symptoms concluded that there is scarce evidence to support the widespread use of hysteroscopic surgery in the general subfertile population [8]. According to the American Society for Reproductive Medicine (ASRM), hysteroscopy is the definitive method for the diagnosis and treatment of intrauterine pathology. As it is also the most costly and invasive method for uterine cavity evaluation, it should be reserved for further evaluation and treatment of abnormalities defined by less invasive methods such as HSG and sonohysterography [9].

A literature review was performed to explore the available information regarding the role of hysteroscopy in the evaluation and management of the infertile female. The literature was reviewed also to ascertain evidence that treatment of these uterine abnormalities (intrauterine adhesions, uterine septa, fibroids, Mullerian anomalies, and endometrial polyps) improves fertility. A MEDLINE search using the key words or combinations of the key words “hysteroscopy, infertility, intrauterine adhesions, uterine septa, fibroids, Mullerian anomalies and endometrial polyps” was performed to identify the relevant publications available by November 15, 2013. No language restriction was applied.

## 2. Fibroids

The mechanisms whereby submucous leiomyomas impact fertility remain unclear. The interference of fibroids on fertility largely depends on their location. Submucous fibroids interfere with fertility and should be removed in infertile patients, regardless of the size or the presence of symptoms [3, 4]. In experienced hands, hysteroscopic myomectomy is minimally invasive, safe, and effective [10].

Hysteroscopy appears unnecessary when the uterine cavity contour is normal. On the other hand, when HSG reveals a filling defect in the uterine cavity, sonohysterography (SIS) or office hysteroscopy can more precisely define the location and attachment of the lesion and determine whether a submucous myoma is amenable to hysteroscopic myomectomy [11]. Despite high-quality evidence from a Cochrane systematic review [12] demonstrating that SIS and hysteroscopy are equivalent for the diagnosis of submucous fibroids, with both being superior to transvaginal ultrasonography (TVUS), the American Association of Gynecologic Laparoscopists (AAGL) [13] recommends that magnetic resonance imaging (MRI) is superior to other imaging and endoscopic techniques in characterizing the relationship of submucous leiomyomas with the myometrium and uterine serosa. The AAGL (2012) [13] considers HSG less sensitive and specific when submucous myomas are concerned. Hysteroscopy revealed high sensibility, specificity, and accuracy in the diagnosis of submucous fibroids and a good correlation with histological diagnosis [14].

The search for randomized controlled trials (RCT) on the treatment of submucous myomas in infertile women retrieved one article [15]. In this prospective randomized matched control trial, 215 women with unexplained primary infertility and with ultrasonographically diagnosed submucous fibroids were enrolled. Women in the study group had a better possibility of becoming pregnant after hysteroscopic myomectomy with a relative risk of 2.1 (95% confidence interval, 1.5–2.9). No difference in pregnancy rates was observed according to fibroid size, number, and location in both groups.

Klatsky et al. (2008) [16] examined the published relationship between uterine fibroids and reproductive outcomes. Submucosal fibroids had the strongest association with lower ongoing pregnancy rates (odds ratio, 0.5; 95% confidence interval, 0.3–0.8) apparently due to decreased embryo implantation. They concluded that, despite the relatively small number of patients studied, there is strong evidence favouring hysteroscopic myomectomy in women before undergoing ART.

Bosteels et al. (2010) [8] performed a systematic review in order to examine the effectiveness of the hysteroscopic removal of submucous fibroids and other intrauterine lesions in subfertile women without other gynaecological symptoms. In patients with one fibroid structure smaller than 4 cm, there was a marginally significant benefit from myomectomy when compared with expectant management (RR (1/4) 1.9; 95% CI: 1.0–3.7).

Pritts et al. (2009) [17] published a systematic literature review and meta-analysis of existing controlled studies regarding the effect of fibroids on fertility and of myomectomy in improving outcomes. They concluded that fertility outcomes are decreased in women with submucosal fibroids and removal seems to confer benefit in terms of pregnancy rates.

A recent Cochrane review [3] tried to assess the effects of the hysteroscopic removal of submucous fibroids in women with otherwise unexplained subfertility or prior to intrauterine insemination, in vitro fertilization (IVF), or intracytoplasmic sperm injection (ICSI). In women with otherwise unexplained subfertility and submucous fibroids, there is no evidence of benefit with hysteroscopic myomectomy compared to regular fertility-oriented intercourse during 12 months for clinical pregnancy (odds ratio (OR) 2.4, 95% confidence interval (CI) 0.97 to 6.2, and *P* = 0.06, 94 women) and miscarriage (OR 1.5, 95% CI 0.47 to 5.0, and *P* = 0.47, 94 women). Nonetheless, the quality of the evidence considered was very low.

According to the ASRM (2008) [11], hysteroscopic myomectomy is indicated for intracavitary myomas and submucous myomas having at least 50% of their volume within the uterine cavity. In infertile women and those with recurrent pregnancy loss, myomectomy should be considered only after a thorough evaluation has been completed. The question of when to advise removal of a fibroid in the infertile female remains a clinical dilemma and conclusions based upon the available literature are still problematic [17].

## 3. Mullerian Anomalies

The prevalence of congenital uterine anomalies in women with reproductive failure remains unclear, largely due to the different diagnostic criteria adopted, heterogeneity of study designs, and selection bias. Thus the current literature regarding the frequency and probable causes of infertility among women with congenital uterine anomalies is insufficient to allow any robust conclusions to be drawn [18]. It appears that women with a history of miscarriage or miscarriage and infertility have higher prevalence of congenital uterine anomalies compared with the unselected population [19].

Although HSG remains a useful screening tool for the diagnosis of a normal or abnormal uterine cavity, showing a good sensitivity for diagnosing uterine malformations, it cannot reliably differentiate between different types of congenital uterine anomalies not allow appropriate classification [5].

Saravelos et al. (2008) [20] reviewed the medical literature in order to assess the diagnostic accuracy of different methodologies and estimate the prevalence of congenital uterine anomalies in women with infertility and recurrent miscarriage. They concluded that the most accurate diagnostic procedures were combined hysteroscopy and laparoscopy, sonohysterography (SIS), and possibly three-dimensional ultrasound (3D US). Despite the fact that the gold standard in the diagnosis of uterine anomalies is the combined application of laparoscopy and hysteroscopy, one has to bear in mind that it is mainly based on the subjective impression of the clinician performing them.

Two-dimensional ultrasound (2D US) and hysterosalpingography are less accurate and are thus inadequate for diagnostic purposes. 2D US provides objective measurable information for the cervix, the uterine cavity, the uterine wall, and the external contour of the uterus with the advantage of being a low-cost noninvasive method, but its accuracy highly depends on the experience of the examiner. 3D US, on the other hand, offers detailed information on uterine anatomy and is a promising option in the diagnosis of uterine anomalies with a very high accuracy rate. It is however more expensive and requires a skilled examiner. These authors considered hysteroscopy alone as an accurate test while MRI has unclear diagnostic accuracy. Preliminary studies however suggest that MRI is a relatively sensitive tool. As hysteroscopy does not allow evaluation of the external contour of the uterus, some may consider it as a suboptimal test [19].

The unicornuate uterus is an uncommon anomaly, which may be associated with relatively poor reproductive outcome depending on a number of factors such as variations in the vascular contribution from the uterine artery and uteroovarian artery of the contralateral side, extent of the reduction of muscular mass of a unicornuate uterus, degree of cervical competence, and presence and extent of coexistent pelvic disease such as endometriosis. The rudimentary horn can be removed by laparotomy or laparoscopy. The bicornuate uterus is a common congenital anomaly and is associated with good reproductive outcomes. Uterus didelphys has a relatively good prognosis for achieving pregnancy when compared with other uterine anomalies [4].

The septate uterus is the most common structural uterine anomaly associated with the highest incidence of reproductive failure [4]. Hysterosalpingography (HSG) may reveal two hemicavities, without visualization of the uterine fundus, and it may be indistinguishable from a bicornuate uterus. In this setting, TVUS is more accurate (100% sensitivity and 80% specificity) [21]. Ghi et al. (2009) [22] suggest that 3D US is extremely accurate for the diagnosis and classification of congenital uterine anomalies and may conveniently become the only mandatory step in the assessment of the uterine cavity in patients with a history of recurrent miscarriage. Although 3D US has been used in the diagnosis of septate uterus, only a few studies are available with no definite conclusion on its role in the identification of the uterine septum [20–22]. Combining diagnostic modalities can improve diagnostic accuracy, but concurrent hysteroscopy and laparoscopy remain the gold standard for diagnosing the septate uterus [4].

Most studies of metroplasty for a septate uterus combine women with recurrent miscarriage and infertility, and no study has been published that randomizes infertile women to treatment versus no treatment. For this reason controversy exists as to whether infertile women should undergo metroplasty [4]. Hysteroscopic metroplasty in women with septate uterus and unexplained infertility could improve clinical pregnancy rate and live birth rate in patients with otherwise unexplained infertility [23, 24].

Hysteroscopic metroplasty in women with recurrent miscarriage and a septate uterus is being performed in many countries to improve reproductive outcomes in this setting. However, only noncontrolled studies suggesting a positive effect on pregnancy outcomes have been performed so far. Nonetheless, these studies are biased due to the fact that the participants with recurrent miscarriage treated by hysteroscopic metroplasty served as their own controls. No randomized controlled trial evaluating the effectiveness and possible complications of hysteroscopic metroplasty has been published so far [25].

The prevalence of the arcuate uterus in women with recurrent pregnancy loss (RPL) is 12.2%, whereas in the general/infertile population it is 3,8%. Such high prevalence in the RPL population suggests a causal relation between this type of uterine anomaly and RPL [20].

## 4. Endometrial Polyps

Endometrial polyps are benign, localized overgrowths of endometrium. They are commonly identified during the investigation for abnormal uterine bleeding and infertility. Little is known about the association between endometrial polyps and fertility. The gold standard for diagnosis is hysteroscopy and hysteroscopic polypectomy remains the mainstay of management [14]. Malignancy arising in polyps is uncommon, and specific risks of malignancy include increasing age and postmenopausal bleeding. Management may be conservative, with up to 25% of polyps regressing, particularly if less than 10 mm in size. Unfortunately, there is a paucity of good quality evidence in the literature on the diagnosis and management of this common gynecologic disease [25]. Polyps can distort the endometrial cavity, may have a detrimental effect on endometrial receptivity, and increase the risk of implantation failure [26].

Stamatellos et al. (2008) [27] evaluated 83 women who met the following criteria: age under 35 years, at least 12 months of infertility, from 3 to 8 months of menstrual disorders (intermenstrual bleeding or spotting, menometrorrhagia or menorrhagia), and from 3 to 18 months of followup with attempts to conceive after hysteroscopic polypectomy. Apparently, the endometrial polyp/polyps appeared to be the only reason to explain their infertility after couple infertility workup. Pregnancy (61.4%) and delivery (54,2%) at term rates increased after the procedure. There was no statistical difference in fertility rates between patients having polyps < or =1 cm and patients having >1 cm polyps or multiple polyps.

A recent Cochrane review [3] tried to assess the effect of hysteroscopic polypectomy on the results of intrauterine insemination (IUI). Apparently, the hysteroscopic removal of polyps prior to IUI increases the odds of clinical pregnancy compared to diagnostic hysteroscopy and polyp biopsy only (OR 4.4, 95% CI 2.5 to 8.0, and *P* < 0.00001).

Implantation and clinical pregnancy rates were statistically significantly increased after hysteroscopic polypectomy in a group of women with recurrent implantation failure after IVF [27].

In conclusion, it appears that polypectomy prior to IUI or IVF (even I cases with previous implantation failure) increases the chances of pregnancy.

## 5. Assisted Reproductive Technology (ART)

Studies evaluating the effect of office hysteroscopy (OH) on pregnancy rate in patients undergoing IVF have been published. Following recurrent IVF failure there is some evidence of benefit from hysteroscopy in increasing the chance of pregnancy in the subsequent IVF cycle, both in those with abnormal and normal hysteroscopic findings [8, 28].

Lorusso et al. (2008) [29] suggest that hysteroscopy as a routine infertility examination should be performed in all cases, owing to the elevated incidence of hysteroscopic pathological findings in these women. Performing OH before IVF-embryo transfer, however, is of no significant value in improving pregnancy outcomes.

Fatemi et al. (2010) [6] tried to assess, by screening office hysteroscopy, the prevalence of unsuspected intrauterine abnormalities in an asymptomatic population of IVF patients, in whom TVS had not revealed any pathology. The observed prevalence of unsuspected intrauterine abnormalities in asymptomatic patients indicated for their first IVF/ICSI treatment appeared to be clearly lower than previously reported (11 versus 20–45%).

A systematic review comparing the outcome of IVF treatment performed in patients who had outpatient hysteroscopy in the cycle preceding their IVF treatment with a control group in which hysteroscopy was not performed was conducted. The results of five studies showed evidence of benefit from outpatient hysteroscopy in improving the pregnancy rate in the subsequent IVF cycle [29].

Karayalcin et al. (2010) [30] reported on a total of 2500 consecutive office-based diagnostic hysteroscopies in an IVF population enrolled prospectively prior to treatment. Endometrial pathology on hysteroscopy which may have impaired the success of IVF was identified in 22.9%.

Karayalçın et al. (2012) [31] enrolled 1258 patients attending an IVF clinic with normal hysteroscopic findings in an attempt to establish the impact of timing of office hysteroscopy before embryo transfer on pregnancy rate. The implantation, pregnancy, and clinical pregnancy rates were significant when office hysteroscopy was performed 50 days or less before embryo transfer.

Another recent study included 157 women with a history of recurrent IVF failures (two or more) who underwent hysteroscopy (diagnostic or operative, as appropriate) to evaluate the endometrial cavity. Abnormal hysteroscopic findings were found in 44.9% of the patients in this study and 75 women (48.1%) became pregnant following hysteroscopy. Of these pregnancies, 36 occurred in women with corrected endometrial pathology, the majority of which was identified as endometrial polyps [28] (Cenksoy et al., 2013).

The safety and diagnostic value of hysteroscopy before IVF were examined in 217 infertile women. In 69 women (31.8%), hysteroscopy identified intrauterine lesions (polyps, septa, submucosal leiomyomas, or synechiae) that led to operative hysteroscopy. The authors concluded that diagnostic hysteroscopy presents significantly higher sensitivity than TVS and HSG in the diagnosis of intrauterine lesions. Thereby, diagnostic hysteroscopy should be performed before IVF in all patients, including women with normal TVS and/or HSG findings, because a significant percentage of them have undiagnosed uterine disease that may adversely affect the success of fertility treatment [32].

The benefit of hysteroscopic surgery was further corroborated in a retrospective matched control study by Tomaževič et al. (2010) [33]. These authors evaluated the influence of septate, subseptate, and arcuate uterus on pregnancy and live birth rates in 2481 in conventionally stimulated IVF/intracytoplasmic sperm injection (ICSI) cycles. Pregnancy rates after embryo transfer before hysteroscopic surgery were significantly lower, both in women with subseptate and septate uterus and in women with arcuate uterus compared with controls. When live birth rates were considered, differences were more evident. The differences disappeared upon hysteroscopic resection [33].

## 6. Conclusion

The position of hysteroscopy in the management of the infertile female remains under debate. Although a variety of studies demonstrate that the procedure is well tolerated and effective in the treatment of intrauterine pathologies, there is no consensus on the effectiveness of hysteroscopic surgery in improving the prognosis of subfertile women.

There are not enough prospective randomized trials to clearly demonstrate that surgical removal of all intrauterine abnormalities improves fertility or IVF outcomes. However, published observational results suggest a benefit for resection of submucosal leiomyomas, adhesions, and at least a subset of polyps in increasing pregnancy rates.

More randomized controlled studies with adequate controls are needed to substantiate the effectiveness of the hysteroscopic removal of suspected endometrial polyps, submucous fibroids, uterine septum, or intrauterine adhesions in women with unexplained subfertility or prior to assisted reproductive technology (IUI, IVF, or ICSI).
